# Thermal convection in nanofluids for peristaltic flow in a nonuniform channel

**DOI:** 10.1038/s41598-022-16600-w

**Published:** 2022-07-25

**Authors:** Sharifah E. Alhazmi, Ali Imran, Muhammad Awais, Mazhar Abbas, Weaam Alhejaili, Haneen Hamam, Awatif Alhowaity, Asif Waheed

**Affiliations:** 1 Mathematics Department, Al-Qunfudah University College, Umm Al-Qura University, Mecca, Kingdom of Saudi Arabia; 2 Department of Mathematics, COMSATS University Islamabad, Attock Campus, Kamra Road, Attock, 43600 Pakistan; 3 Department of Mathematical Sciences, College of Science, Princess Nourah bint Abdulrahman University, P.O. Box 84428, Riyadh, 11671 Saudi Arabia; 4 Mathematics Department, Umm Al-Qura University, Makkah, Kingdom of Saudi Arabia; 5 Department of Mathematics, College of Science and Arts at Alkamil, University of Jeddah, Jeddah, Saudi Arabia

**Keywords:** Biophysics, Mathematics and computing

## Abstract

A magneto couple stress nanofluid flow along with double diffusive convection is presented for peristaltic induce flow through symmetric nonuniform channel. A comprehensive mathematical model is scrutinized for couple stress nanofluid magneto nanofluids and corresponding equations of motions are tackled by applying small Reynolds and long wavelength approximation in viewing the scenario of the biological flow. Computational solution is exhibited with the help of graphical illustration for nanoparticle volume fraction, solutal concentration and temperature profiles in MATHEMTICA software. Stream function is also computed numerically by utilizing the analytical expression for nanoparticle volume fraction, solutal concentration and temperature profiles. Whereas pressure gradient profiles are investigated analytically. Impact of various crucial flow parameter on the pressure gradient, pressure rise per wavelength, nanoparticle volume fraction, solutal concentration, temperature and the velocity distribution are exhibited graphically. It has been deduced that temperature profile is significantly rise with Brownian motion, thermophoresis, Dufour effect, also it is revealed that velocity distribution really effected with strong magnetic field and with increasing non-uniformity of the micro channel. The information of current investigation will be instrumental in the development of smart magneto-peristaltic pumps in certain thermal and drug delivery phenomenon.

## Introduction

The nonlinear flows involving peristalsis phenomenon are achieving new horizons in view of their core applications in the transport processes in the domains of chemical and biomedical science (bile ducts, esophagus, uterine cavity etc.), bio plasma transport etc. Mathematical modelling involving formulation of the peristaltic processes has tremendous applications in engineering and industries. For-instance Ellahi^1^ investigated characteristics of heat and mass transport for the peristaltic flow in a non-uniform rectangular duct. Authors have studied and presented several important results regarding the bioheat transfer model due to its applications in thermotherapy and human thermoregulation system. Sreenadh^2^ studied the peristaltic flow of conducting nanofluids in a non-symmetric channel with slip effects of velocity, temperature and concentration. Sharma^3^ analyzed the electro-osmosis based peristaltic flow of nanofluid. Authors have investigated the double diffusive convection phenomenon. Awais et al.^4^ studied the second law properties and endoscopy applications for hydro magnetic peristaltic flow of nanofluid. Slippage phenomenon along with permeable surface on peristaltic flow of hydro magnetic Ree Eyring nanofluid has been studied by Tanveer and Malik^5^. They have investigated the comprehensive study in a curved channel with porous media by utilizing the modified Darcy’s law. Riaz et al.^6^ analyzed the peristaltic propulsion of Jeffery nanomaterial. They have studied the heat transfer characteristics within duct with dynamic wall and permeable medium. Heat transfer properties of biological nanoliquid flow dynamics through ductus efferentus have been presented by Imran et al.^7^. Hydro magnetic analysis and heat transfer effects through ductus efferentus involving variable viscosity phenomenon has been analyzed by Imran et al.^8^. Thermal and micro rotation process involving Cu-CuO/blood nanoparticles in a microvascular geometry has been analyzed by Tripathi et al.^9^. Blood flow of hydro magnetic non-Newtonian nanomaterial involving heat transfer and slip effect has been analyzed by Aasma et al.^10^. Qureshi et al.^11^ analyzed impacts of radially magnetic field axioms in a peristaltic flow with internally generated heat phenomena. Parveen et al.^12^ studied thermophysical axioms of chemotactic microorganisms in bio-convective peristaltic flow dynamics of nanoliquid with slipp and Joule heating effects. Some latest studies on the recent development on peristaltic phenomenon are presented in Refs.^7,8,13–21^.

Divya et al.^22^ presented the biological dynamics of a Casson fluid within a non-uniform channel along with radially applied magnetic field. Analytical investigation for unsteady dynamics of a Rabinowitsch fluid through afore mentioned geometry is revealed for variable liquid properties^23^.

Note that with the advent of modern computers, numerical computing (one of the latest technique) to perform highly parallel computing involving difficult navigation and recognition tasks have been utilized by many researcher to tackle the complex problems. The approximate solutions have lost some of their significance since recently developed numerical algorithms are available to tackle the progressively realistic and complex problems. It is due to the fact the a computed numerical result, requiring nominal effort with significant precision is mostly useful for scientist, engineers and applied mathematician who may acquire the core insight of the problem easily. Researcher have employed numerical computing recently in a variety of domains. For-instance Singh et al.^24^ computed numerical results of micropolar fluid. Authors have considered the flow situation over a stretching surface with chemical reaction along with melting heat transfer. They have employed the Keller-Box method to compute the results. Pandey^25^ experimentally investigated with the aid of numerical simulation thermal and flow properties of a shear-thinning non-Newtonian fluid in a varyingly heated cavity. Awais et al.^26^ examined the fluid rheology of bioconvective nanofluid possessing dynamic microorganisms with the help of numerical computations and revealed heat and mass transfer phenomena. Salmi et al.^27^ also presented numerical study with heat and mass transfer development in Prandtl fluid magnetohydrodynamic flow using Cattaneo-Christov heat flux theory. Awais et al.^28^ analyzed generalized MHD impacts in a Sakiadis flow of polymeric nano liquids. In this communication our objective is to explore further in the regime of biological flow computations via numerical computing. The tendency of numerical computing to deal with the complex nature of biological/peristaltic flow model motivates the authors to investigate the double-diffusive convection process via thermal and concentration properties for the non-uniform biological geometry. Flow dynamics of couple-stress nanomaterial under the application of magnetic field are computed. Mathematical modellings have been performed and dataset is computed and comprehensive studies for emerging physical quantities are performed to examine the outcome of each term. Tackling such bio-mathematical problems are important to modernize the diagnostic processes of several issues arise in peristaltic phenomenon.

Further physiological impacts are explored for the biological flow of rheological fluid through inclined geometries by^29–33^. Hayat et al.^34^ elaborated the effect of thermal radiation along with MHD for the Jeffrey fluid. A mathematical investigation for the peristaltic flow of couple-stress fluid in a transverse non-symmetric channel with bon-isothermal scenario has been reported^35^. One may find related heat transfer analysis for peristaltically induce flow for couple stress fluid^21,36,37^.

## Mathematical interpretation of physical problem

**Figure 1:**
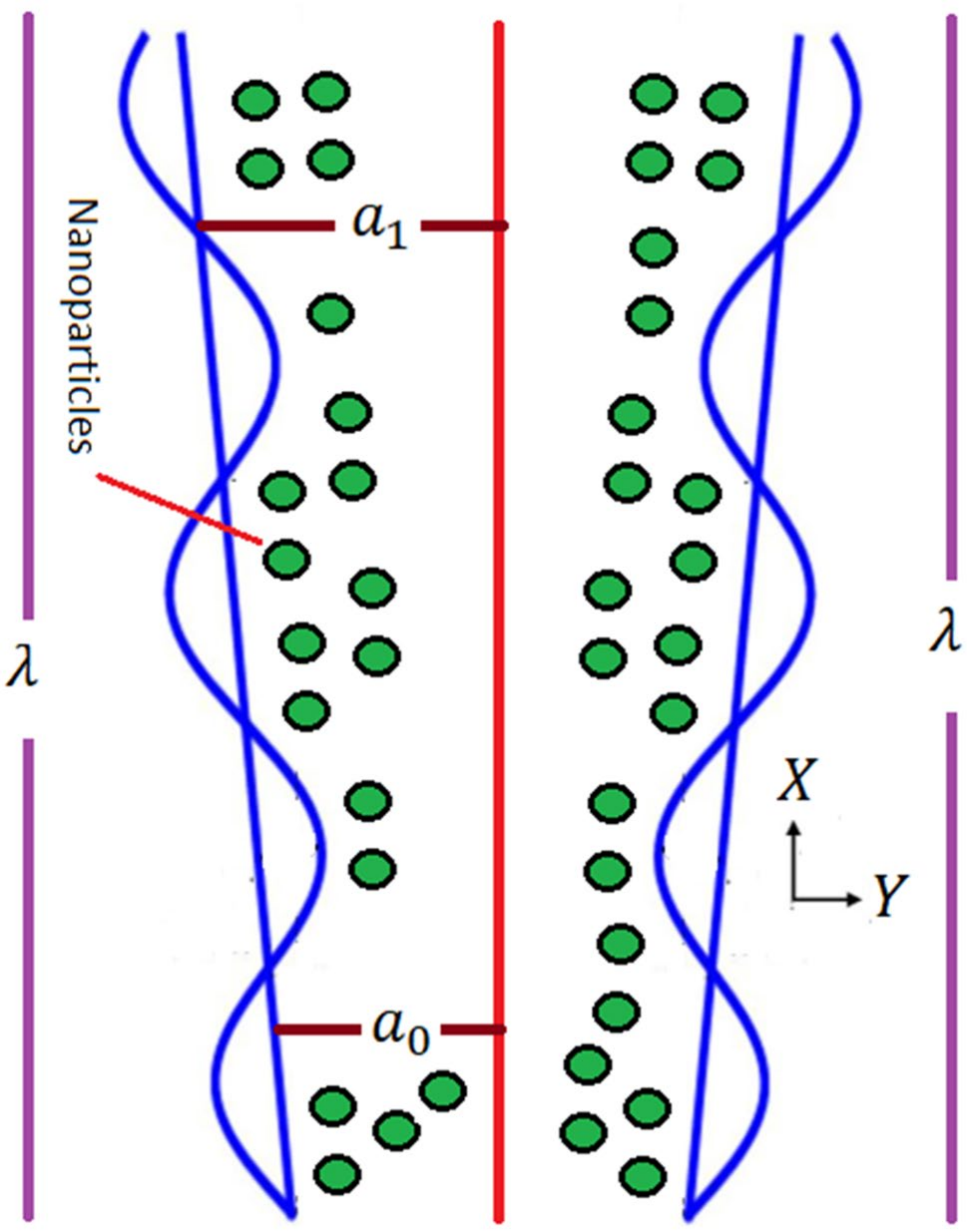
Problem's geometry.

Let us emphasis on the flow dynamics of electrically conducting couple stress fluid in a nonuniform channel in an incompressible MHD flow. Waves pass alongside the channel walls, causing flow. Assume we have a rectangular coordinate system with the X-axis aligned with wave propagation and the Y-axis parallel to it. The induced magnetic field is led by a continuous magnetic area of strength acting in a transverse direction. The magnetic field in general is defined as (Fig. 1)

$$H^{ + } (\tilde{h}_{X} (X,\;Y,\;t),H_{0} + \tilde{h}_{Y} (X,\;Y,\;t),\;0).$$

The following is the geometrical description of the physical problem^21–33,33–37^:

1 $$H(X,t) = b\sin \left( {\frac{2\pi }{{\lambda_{1} }}(X - ct)} \right) + a(X),$$

with $$a(X) = a_{0} + a_{1} X$$.

Within equation the parameters $$\lambda ,$$$$a_{0}$$, t, $$a$$, b, c, represents wavelength, half width at inlet, time, half breadth of channel, wave amplitude, speed of the wave and respectively.

The continuity, momentum, energy, concentration, and nanoparticle volume fraction equations^33,34^ are:

2 $$\left( {\frac{\partial U}{{\partial X}} + \frac{\partial V}{{\partial Y}}} \right) = 0,$$

3 $$\begin{aligned} \rho_{f} \left( {\frac{\partial }{\partial t} + U\frac{\partial }{\partial X} + V\frac{\partial }{\partial Y}} \right)U = & - \frac{\partial P}{{\partial X}} + \mu \left( {\frac{{\partial^{2} U}}{{\partial X^{2} }} + \frac{{\partial^{2} U}}{{\partial Y^{2} }}} \right) \\ & - \eta \left( {\frac{{\partial^{4} }}{{\partial X^{4} }} + \frac{{\partial^{4} }}{{\partial Y^{4} }} + 2\frac{{\partial^{4} }}{{\partial X^{2} \partial Y^{2} }}} \right)U \\ & - \frac{{\mu_{e} }}{2}\left( {\frac{{\partial H^{{ + }{^{2} }} }}{\partial Y}} \right) + \mu_{e} \left( {h_{X} \frac{\partial }{\partial X} + h_{Y} \frac{\partial }{\partial Y} + H_{0} \frac{\partial }{\partial Y}} \right)h_{X} \\ & + g\{ \rho_{f0} (1 - \Theta )\{ \beta_{T} (T - T_{0} ) + \beta_{C} (C - C_{0} )\} \\ & - (\rho - \rho_{f0} )(\Theta - \Theta_{0} )\} . \\ \end{aligned}$$

4 $$\begin{aligned} \rho_{f} \left( {\frac{\partial }{\partial t} + U\frac{\partial }{\partial X} + V\frac{\partial }{\partial Y}} \right)V = & - \frac{\partial P}{{\partial Y}} + \mu \left( {\frac{{\partial^{2} V}}{{\partial X^{2} }} + \frac{{\partial^{2} V}}{{\partial Y^{2} }}} \right) \\ & - \eta \left( {\frac{{\partial^{4} }}{{\partial X^{4} }} + \frac{{\partial^{4} }}{{\partial Y^{4} }} + 2\frac{{\partial^{4} }}{{\partial X^{2} \partial Y^{2} }}} \right)V \\ & - \frac{{\mu_{e} }}{2}(\frac{{\partial H^{{ + }{^{2} }} }}{\partial Y}) + \mu_{e} \left( {h_{X} \frac{{\partial h_{X} }}{\partial X} + h_{Y} \frac{{\partial h_{X} }}{\partial Y} + H_{0} \frac{{\partial h_{X} }}{\partial Y}} \right) \\ & + g\rho_{f0} \{ (1 - \Theta )\{ \beta_{T} (T - T_{0} ) + \beta_{C} (C - C_{0} )\} \\ & - (\rho - \rho_{f0} )(\Theta - \Theta_{0} )\} . \\ \end{aligned}$$

5 $$\begin{aligned} (\rho c)_{f} \left( {U\frac{\partial }{{\partial X}} + V\frac{\partial }{{\partial Y}} + \frac{\partial }{{\partial t}}} \right)T = & \sigma \left( {\frac{{\partial ^{2} }}{{\partial Y^{2} }} + \frac{{\partial ^{2} }}{{\partial X^{2} }}} \right)T \\ & + (\rho c)_{f} \left\{ {D_{B} \left( {\frac{{\partial \Theta }}{{\partial X}}\frac{\partial }{{\partial X}} + \frac{{\partial \Theta }}{{\partial Y}}\frac{\partial }{{\partial Y}}} \right)T} \right. \\ & \left. {\left( {\frac{{D_{T} }}{{T_{0} }}} \right)\left[ {\left( {\frac{{\partial T}}{{\partial X}}} \right)^{{^{2} }} + \left( {\frac{{\partial T}}{{\partial Y}}} \right)^{{^{2} }} } \right]} \right\} + D_{{TC}} \left( {\frac{{\partial ^{2} C}}{{\partial X^{2} }} + \frac{{\partial ^{2} C}}{{\partial Y^{2} }}} \right), \\ \end{aligned}$$

6 $$\left( {\frac{\partial }{\partial t} + U\frac{\partial }{\partial X} + V\frac{\partial }{\partial Y}} \right)C = D_{s} \left( {\frac{{\partial^{2} C}}{{\partial X^{2} }} + \frac{{\partial^{2} C}}{{\partial Y^{2} }}} \right) + D_{TC} \left( {\frac{{\partial^{2} T}}{{\partial X^{2} }} + \frac{{\partial^{2} T}}{{\partial Y^{2} }}} \right),$$

7 $$\left( {\frac{\partial }{\partial t} + U\frac{\partial }{\partial X} + V\frac{\partial }{\partial Y}} \right)\Theta = \left( {\frac{{D_{T} }}{{T_{0} }}} \right)\left( {\frac{{\partial^{2} T}}{{\partial X^{2} }} + \frac{{\partial^{2} T}}{{\partial Y^{2} }}} \right) + D_{B} \left( {\frac{{\partial^{2} }}{{\partial X^{2} }} + \frac{{\partial^{2} }}{{\partial Y^{2} }}} \right)\Theta ,$$

In order to further simplified the flow analysis, the following transformations would be used to examine the flow from laboratory frame of situation to wave frame scenario.

8 $$\begin{aligned} \overset{\lower0.5em\hbox{$\smash{\scriptscriptstyle\frown}$}}{x} & = X - ct,\;\overset{\lower0.5em\hbox{$\smash{\scriptscriptstyle\frown}$}}{u} = U - c,\;\overset{\lower0.5em\hbox{$\smash{\scriptscriptstyle\frown}$}}{y} = Y. \\ \overset{\lower0.5em\hbox{$\smash{\scriptscriptstyle\frown}$}}{v} & = V,p(\overset{\lower0.5em\hbox{$\smash{\scriptscriptstyle\frown}$}}{x} ,\;\overset{\lower0.5em\hbox{$\smash{\scriptscriptstyle\frown}$}}{y} ) = P(X,Y,t),\;\overset{\lower0.5em\hbox{$\smash{\scriptscriptstyle\frown}$}}{C} (\overset{\lower0.5em\hbox{$\smash{\scriptscriptstyle\frown}$}}{x} ,\;\overset{\lower0.5em\hbox{$\smash{\scriptscriptstyle\frown}$}}{y} ) = C(X,Y,t) \\ \overset{\lower0.5em\hbox{$\smash{\scriptscriptstyle\frown}$}}{T} (\overset{\lower0.5em\hbox{$\smash{\scriptscriptstyle\frown}$}}{x} ,\;\overset{\lower0.5em\hbox{$\smash{\scriptscriptstyle\frown}$}}{y} ) & = T(X,Y,t),\;\overset{\lower0.5em\hbox{$\smash{\scriptscriptstyle\frown}$}}{\Theta } (\overset{\lower0.5em\hbox{$\smash{\scriptscriptstyle\frown}$}}{x} ,\;\overset{\lower0.5em\hbox{$\smash{\scriptscriptstyle\frown}$}}{y} ) = \Theta (X,Y,t). \\ \end{aligned}$$

Making use of underneath transformations one may have.

9 $$\begin{aligned} \overset{\lower0.5em\hbox{$\smash{\scriptscriptstyle\frown}$}}{x} & = \frac{x}{{\lambda_{1} }},\;\overline{y} = \frac{y}{{b_{0} }},\;\overset{\lower0.5em\hbox{$\smash{\scriptscriptstyle\frown}$}}{v} = \frac{v}{c},\;\overset{\lower0.5em\hbox{$\smash{\scriptscriptstyle\frown}$}}{u} = \frac{u}{c},\overline{p} = \frac{{b_{0}^{2} p}}{{\mu c\lambda_{1} }},\;\overline{t} = \frac{ct}{{\lambda_{1} }},\;\theta = \frac{{\overset{\lower0.5em\hbox{$\smash{\scriptscriptstyle\frown}$}}{T} - T_{0} }}{{T_{1} - T_{0} }},\;R_{m} = \sigma \mu_{e} b_{0} c, \\ \Omega & = \frac{{\overline{\Theta } - \Theta_{0} }}{{\Theta_{1} - \Theta_{0} }},\;N_{CT} = \frac{{D_{CT} (T_{1} - T_{0} )}}{{(C_{1} - C_{0} )D_{s} }},\;N_{TC} = \frac{{D_{CT} (C_{1} - C_{0} )}}{{\sigma (T_{1} - T_{0} )}},\;\overline{h} = \frac{h}{{b_{0} }},\;\overline{\Phi } = \frac{\Phi }{{H_{0} b_{0} }}, \\ N_{b} & = \frac{{(\rho c)_{p} D_{B} (\Theta_{1} - \Theta_{0} )}}{\sigma },\;N_{t} = \frac{{(\rho c)_{p} D_{T} (T_{1} - T_{0} )}}{{T_{0} \sigma }},\;\lambda = \frac{{\overline{C} - C_{0} }}{{C_{1} - C_{0} }},\;h_{X} = \frac{\partial \Phi }{{\partial y}} \\ \end{aligned}$$

Making use of above quantities in Eqs. (2–7) along with long wavelength and low Reynolds numbers we get.

10 $$- \frac{\partial p}{{\partial x}} + \frac{{\partial^{3} \psi }}{{\partial y^{3} }} - \frac{1}{{k^{2} }}\frac{{\partial^{5} \psi }}{{\partial y^{5} }} + G_{rt} \theta + G_{rc} \lambda - G_{rF} \Omega = 0$$

11 $$\frac{{\partial^{2} \theta }}{{\partial y^{2} }} + N_{TC} \frac{{\partial^{2} \lambda }}{{\partial y^{2} }} + N_{b} \left( {\frac{\partial \theta }{{\partial y}}\frac{\partial \Omega }{{\partial y}}} \right) + N_{t} \left( {\frac{\partial \theta }{{\partial y}}} \right)^{2} = 0,$$

12 $$\frac{{\partial ^{2} \lambda }}{{\partial y^{2} }} + N_{{TC}} \frac{{\partial ^{2} \theta }}{{\partial y^{2} }} = 0$$

13 $$\frac{{\partial^{2} \Omega }}{{\partial y^{2} }} + \frac{{N_{t} }}{{N_{b} }}\frac{{\partial^{2} \theta }}{{\partial y^{2} }} = 0,$$

14 $$\Phi_{yy} = R_{m} (E - \psi_{y} ),$$

15 $$\frac{{\partial^{6} \psi }}{{\partial y^{6} }} - k^{2} \frac{{\partial^{4} \psi }}{{\partial y^{4} }} - k^{2} M^{2} \left( {\frac{{\partial^{2} \psi }}{{\partial y^{2} }}} \right) + k^{2} (G_{rt} \theta_{y} + G_{rc} \lambda_{y} - G_{rF} \Omega_{y} ) = 0,$$

The above-mentioned equations are associated with following boundary conditions

16 $$\theta = 0,\;\Omega = 0,\;\lambda = 0{\text{ on y = 0,}}$$

17 $$\theta = 1,\;\Omega = 1,\;\lambda = 1{\text{ on y}} = {\text{h}},$$

18 $$\Phi = {\text{0 on y}} = h,\;{\text{and}}\quad \frac{\partial \Phi }{{\partial y}} = 0{\text{ at y}} = {0, }$$

19 $$\begin{gathered} \psi = 0{ ,}\frac{{\partial^{2} \psi }}{{\partial y^{2} }} = {0, }\frac{{\partial^{4} \psi }}{{\partial y^{4} }} = {\text{0 on y}} = {0, } \hfill \\ \psi = F,\frac{\partial \psi }{{\partial y}} = { - }{1,}\frac{{\partial^{3} \psi }}{{\partial y^{3} }} = {\text{0 on y}} = {\text{h}}, \hfill \\ \end{gathered}$$

where $${\text{h}} = {\text{1 + m}}_{1} x + \alpha \sin (2\pi x)$$.

where *m*_*1*_ non dimensional width of the inlet, $\alpha$ is the non-uniform width^22,23^ of the channel.

The expression for current density and axial induced magnetic field is defined as.

20 $${\text{J}}_{{\text{z}}} = - \frac{{\partial {\text{h}}_{{\text{X}}} }}{\partial y},\;h_{x} = \frac{\partial \Phi }{{\partial y}}$$

To explore pressure rise per wavelength we may have.

$$\Delta p = \int\limits_{0}^{1} {\frac{{{\text{d}}\overset{\lower0.5em\hbox{$\smash{\scriptscriptstyle\frown}$}}{p} }}{{{\text{d}}x}}{\text{d}}x}$$

## Solution of the problem

Equations (11–13) are tackled numerically in Mathematica, whereas the Eq. (15) after capitalizing exact expressions from Eqs. (21–23) is also solved computationally.

The expression for nanoparticle volume fraction is obtained from Eq. (13) as,

21 $$\Omega = - \;\frac{{N_{t} \left( {c5(N_{CT} N_{TC} - 1)e^{{\frac{{c2yN_{b} }}{{N_{CT} N_{TC} - 1}}}} + c6} \right)}}{{N_{b} }}\; + \;c1\; + \;c2y,$$

Similarly, the expression for solutal concentration is obtained from Eq. (12) as,

22 $$\lambda = c3 + c4y - \left( { - \frac{1}{{e^{{\frac{{c2hN_{b} }}{{ - 1 + N_{{CT}} N_{{TC}} }}}} }} + \frac{{e^{{\frac{{c2yN_{b} }}{{ - 1 + N_{{CT}} N_{{TC}} }}}} }}{{ - 1 + e^{{\frac{{c2hN_{b} }}{{ - 1 + N_{{CT}} N_{{TC}} }}}} }}} \right)N_{{CT}} ,$$

By utilizing Eq. (11) the temperature expression is obtained as,

23 $$\theta = c6 + \frac{{e^{{\frac{{c2yN_{b} }}{{N_{CT} N_{TC} - 1}}}} c5( - 1 + N_{CT} N_{TC} )}}{{c2N_{b} }},$$

The constants c1 through c6 are determined using boundary conditions (16, 17). The following are the values of these constants:

$$\begin{gathered} c1 = 0,\;c2 = - \frac{{ - N_{b} - N_{t} }}{{hN_{b} }}, \hfill \\ c3 = 0,\;c4 = - \frac{{ - 1 - N_{CT} }}{h}, \hfill \\ c5 = \frac{{c2N_{b} }}{{\left( { - 1 + e^{{\frac{{c2hN_{b} }}{{ - 1 + N_{CT} N_{TC} }}}} } \right)( - 1 + N_{CT} N_{TC} )}}, \hfill \\ c6 = - \frac{1}{{ - 1 + e^{{\frac{{c2hN_{b} }}{{ - 1 + N_{CT} N_{TC} }}}} }}, \hfill \\ \end{gathered}$$

## Graphical observations

**Figure 2:**
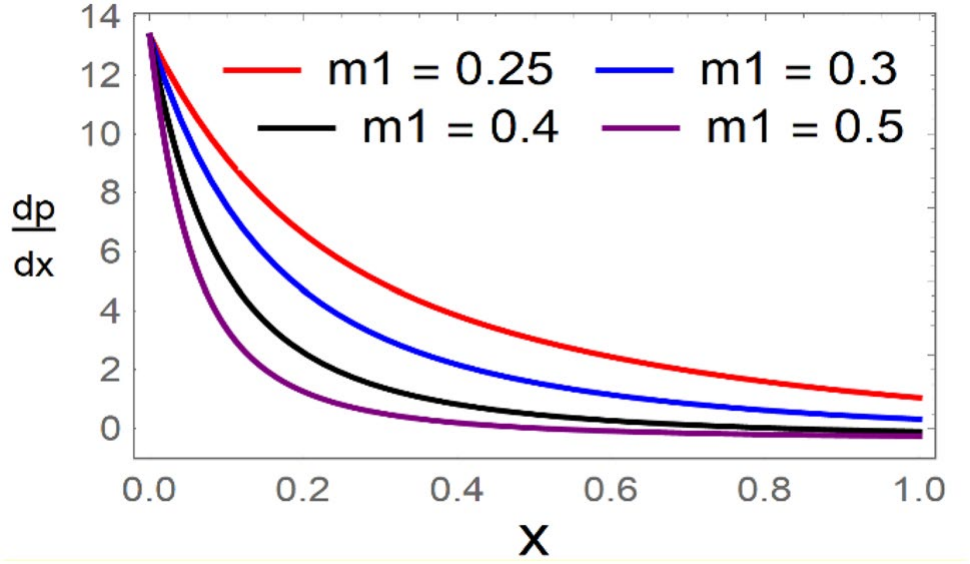
$$\frac{{{\text{d}}p}}{{{\text{d}}x}}$$ profile for m1.

**Figure 3:**
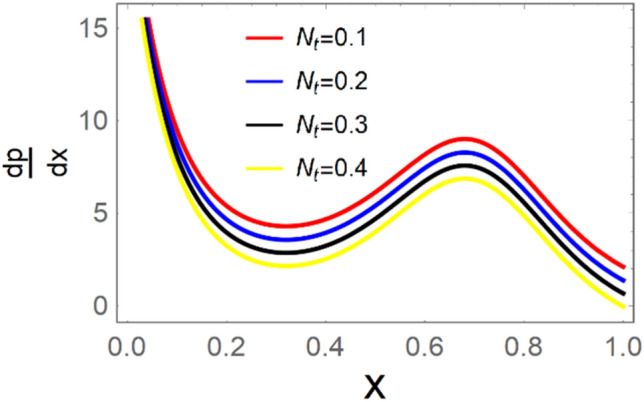
$$\frac{{{\text{d}}p}}{{{\text{d}}x}}$$ profile for *N*_*t*_.

**Figure 4:**
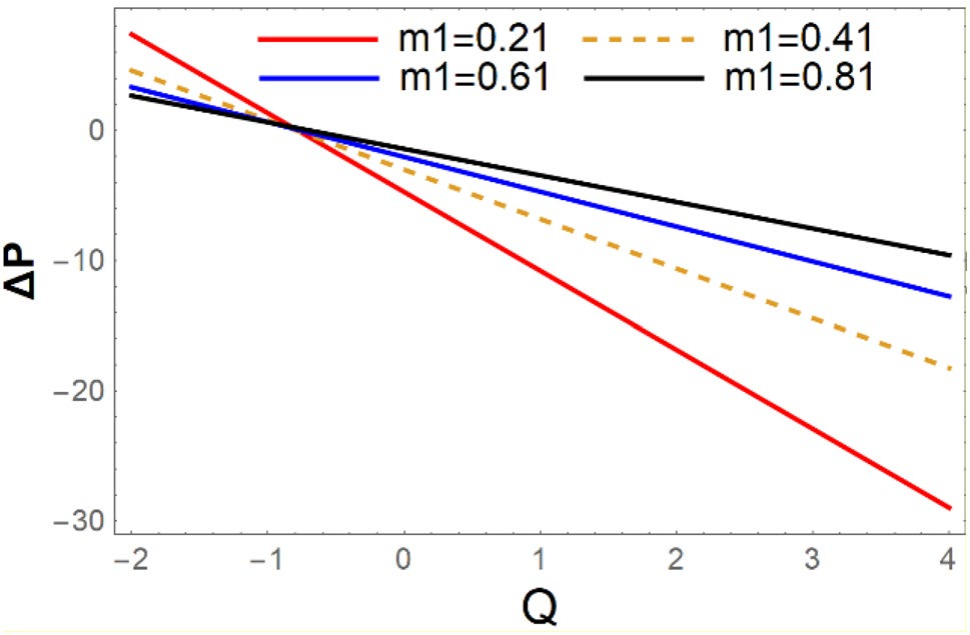
$\Delta p$ impact for varying m1.

**Figure 5:**
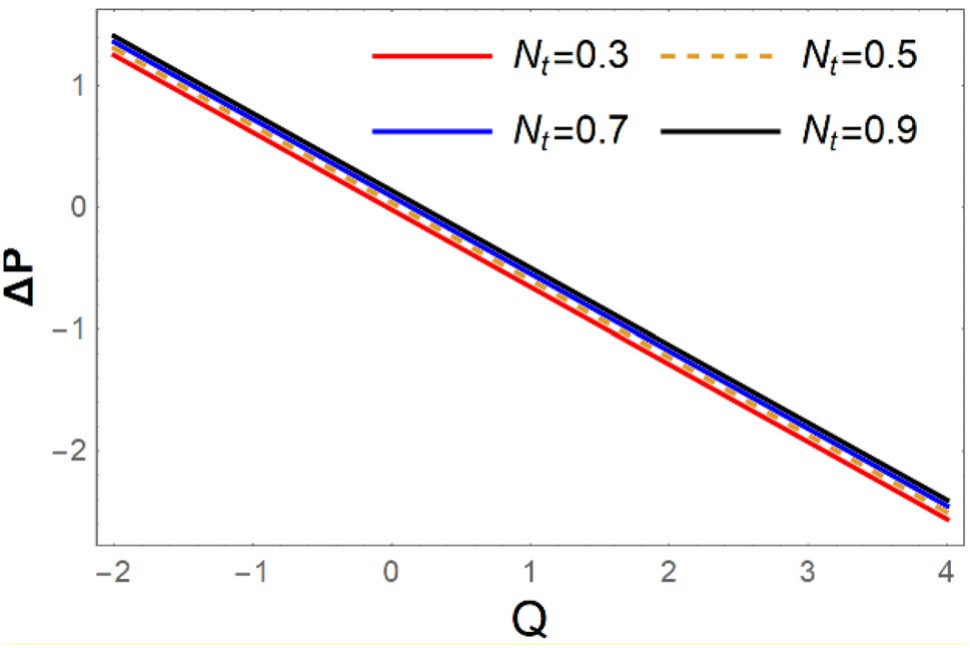
$\Delta p$ impact for varying profile for *N*_*t*_.

**Figure 6:**
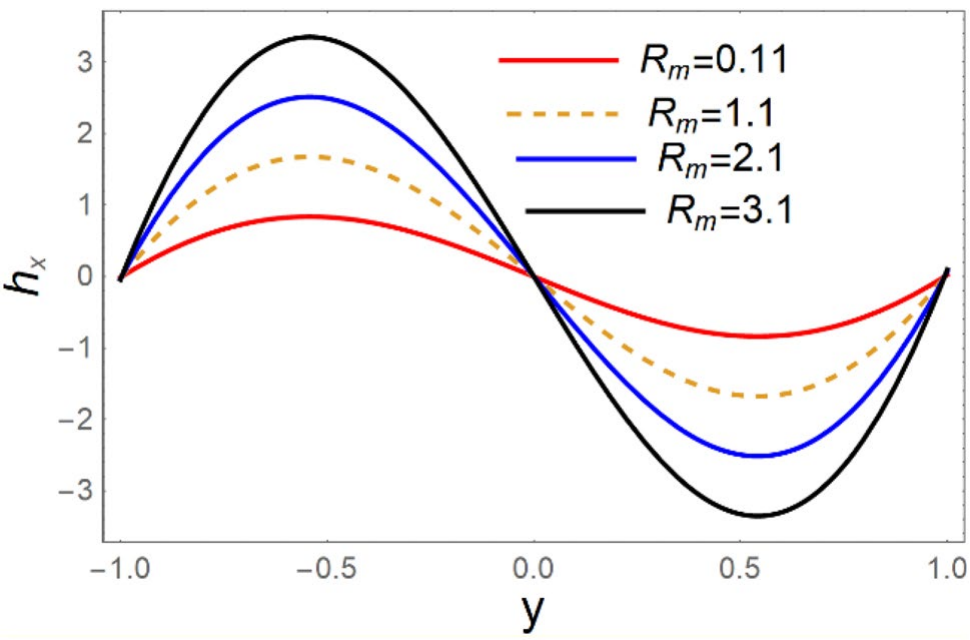
$$h_{x}$$ variations for *R*_*m*_.

**Figure 7:**
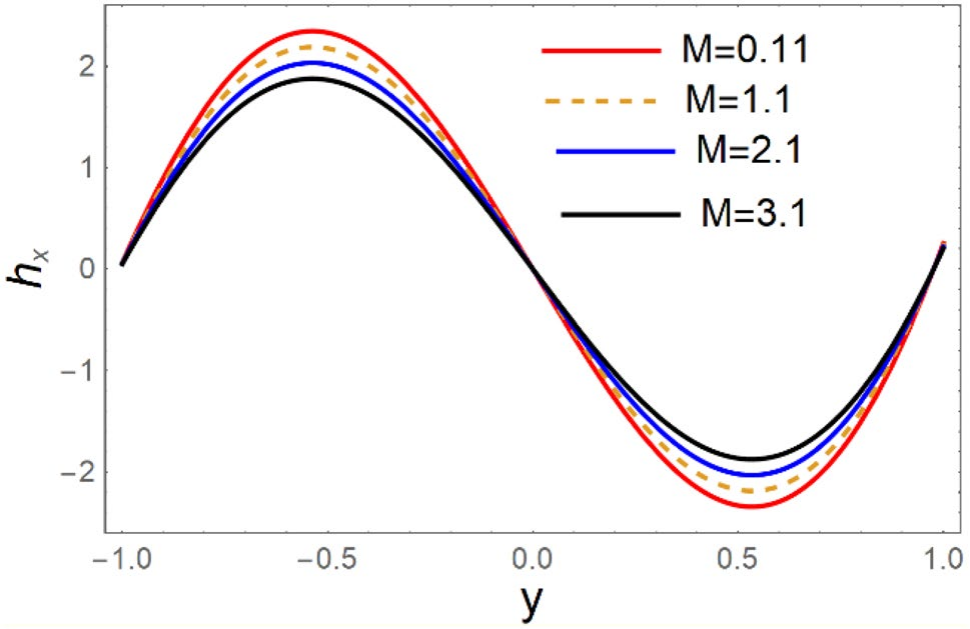
$$h_{x}$$ variations for M.

**Figure 8:**
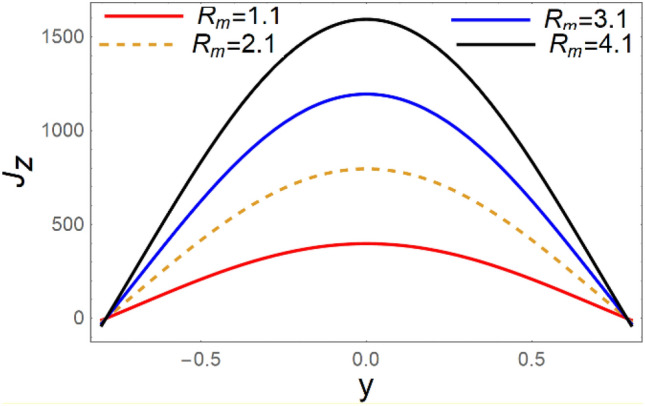
$$J_{z}$$ variations for *R*_*m*_.

**Figure 9:**
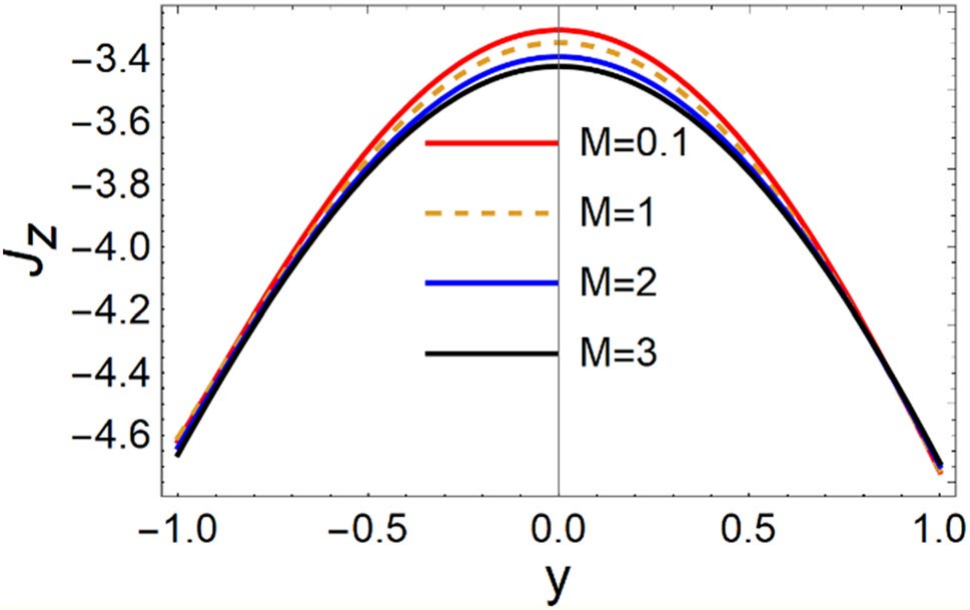
$$J_{z}$$ variations for M.

**Figure 10:**
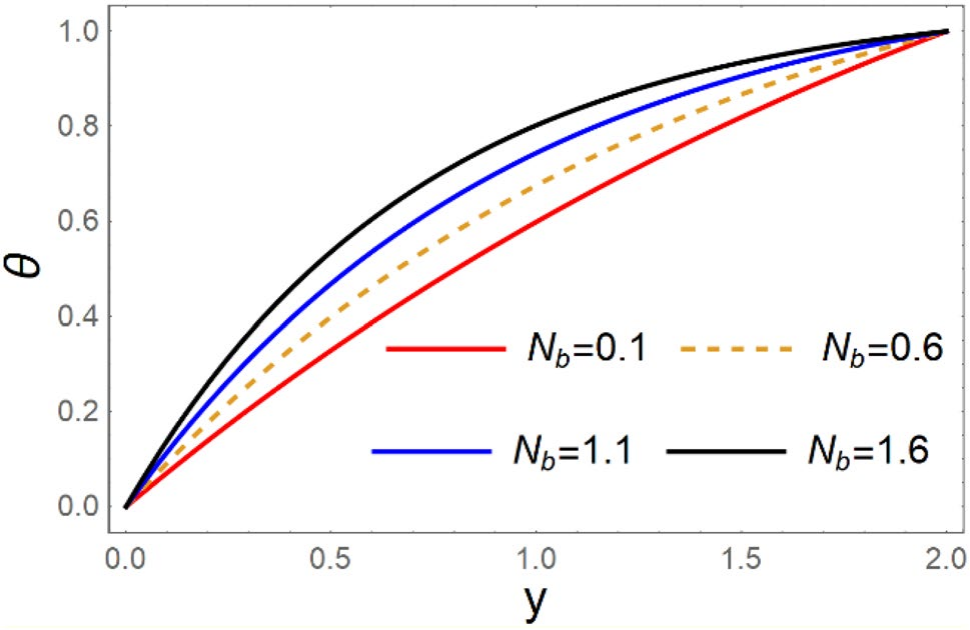
$$\theta$$ variations for $$N_{b}$$.

**Figure 11:**
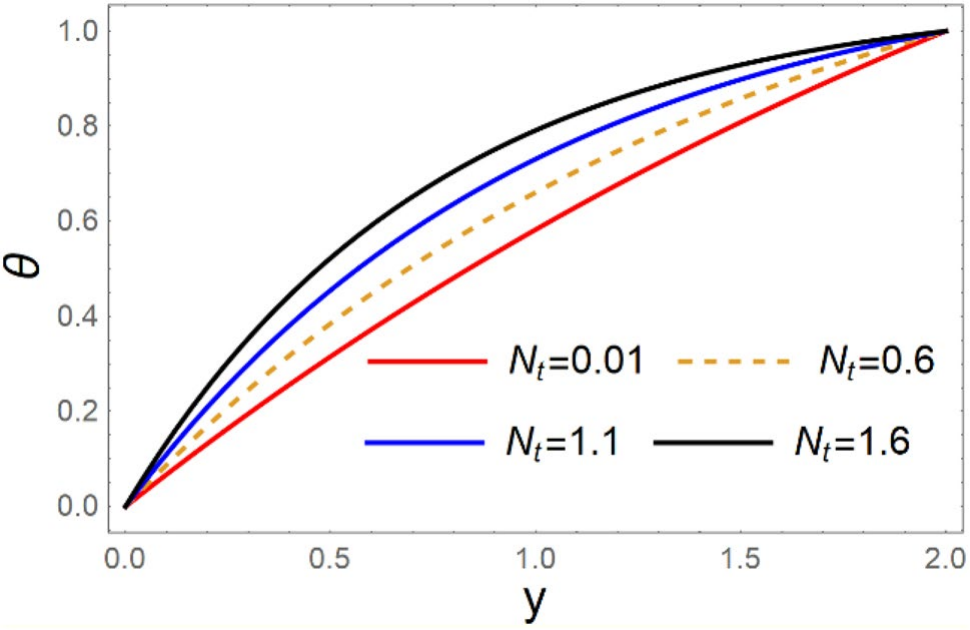
*θ* variations for $$N_{t}$$.

**Figure 12:**
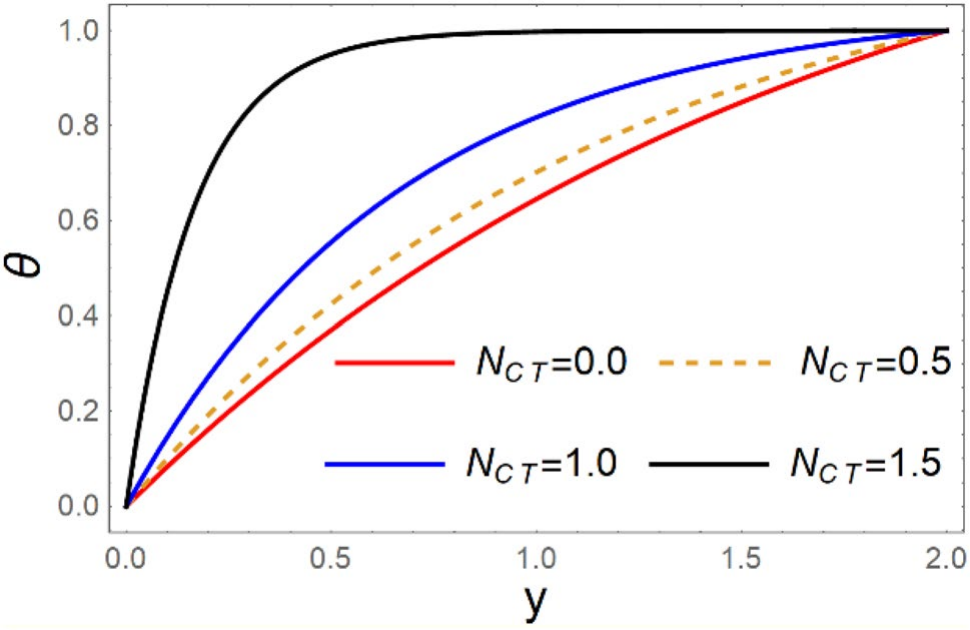
*θ* variations for $$N_{CT}$$.

**Figure 13:**
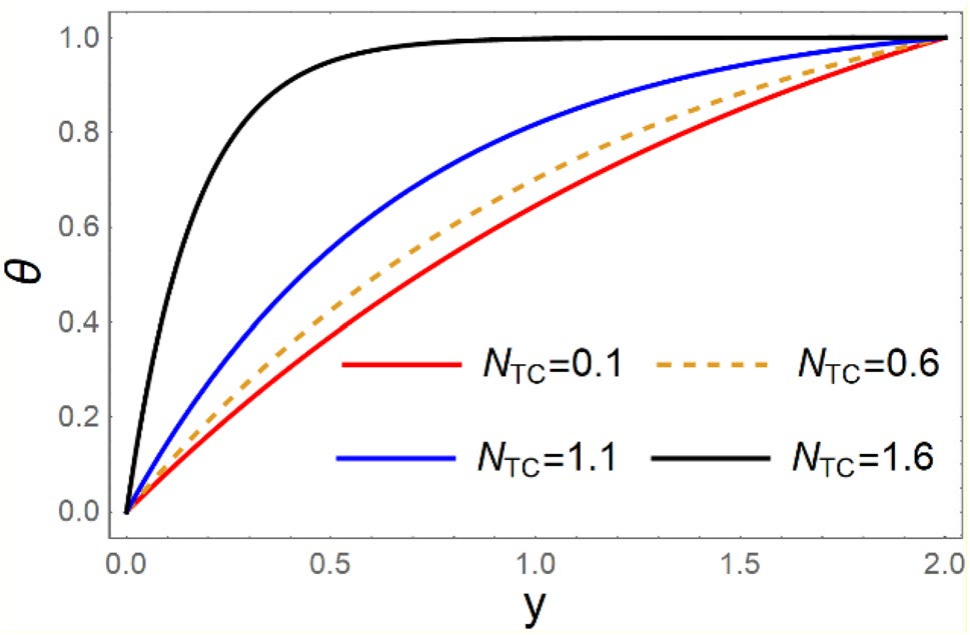
*θ* variations for $$N_{TC}$$.

**Figure 14:**
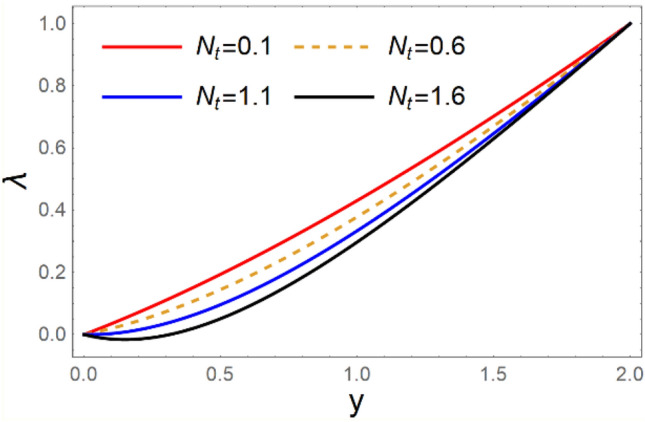
$$\lambda$$ profile for *N*_*b*_.

**Figure 15:**
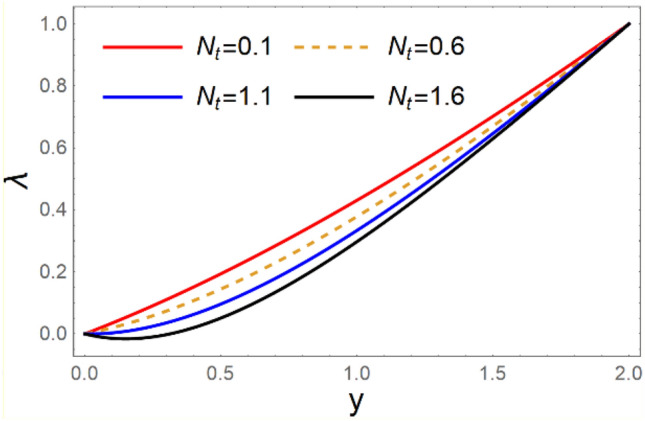
*λ* profile for *N*_*t*_.

**Figure 16:**
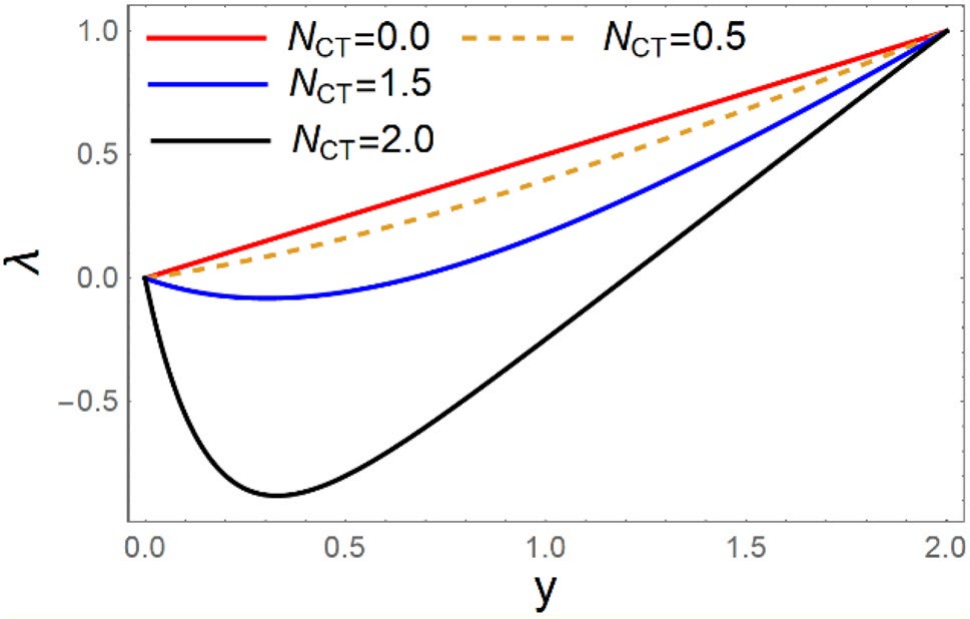
*λ* variations for *N*_*CT*_.

**Figure 17:**
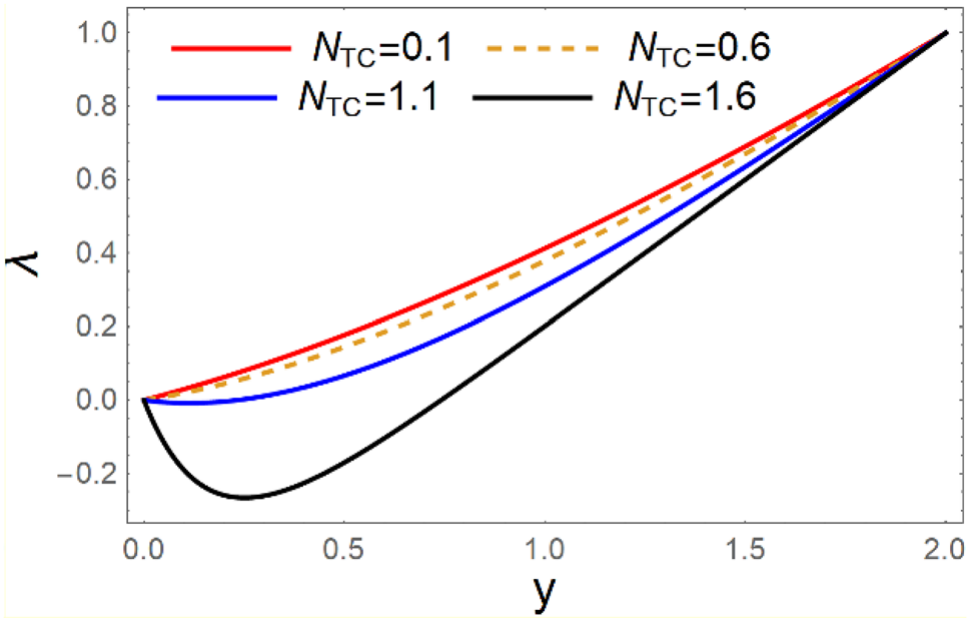
*λ* variations for* N*_*TC*_.

**Figure 18:**
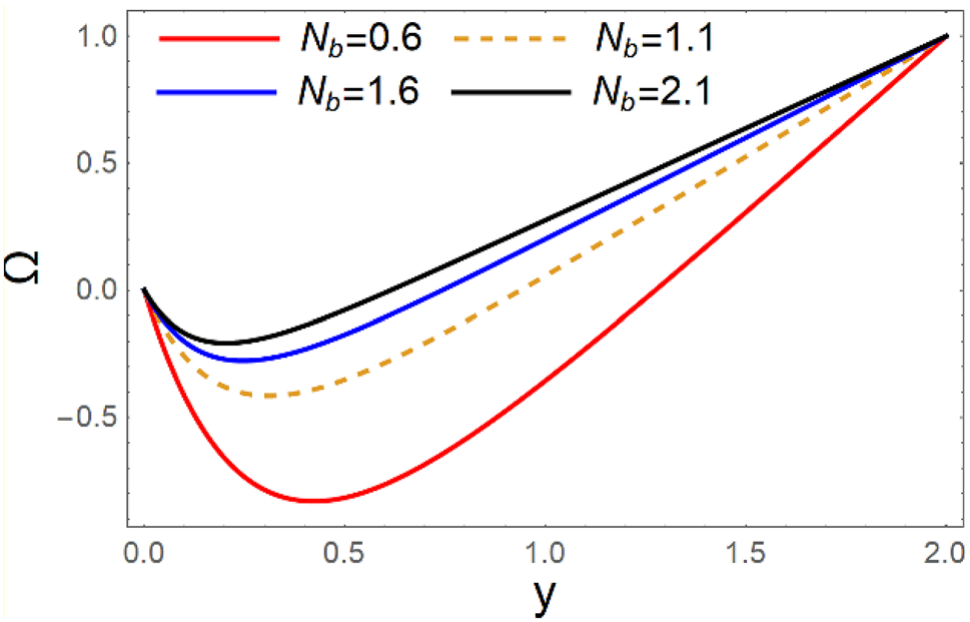
Ω variations for* N*_*b*_.

**Figure 19:**
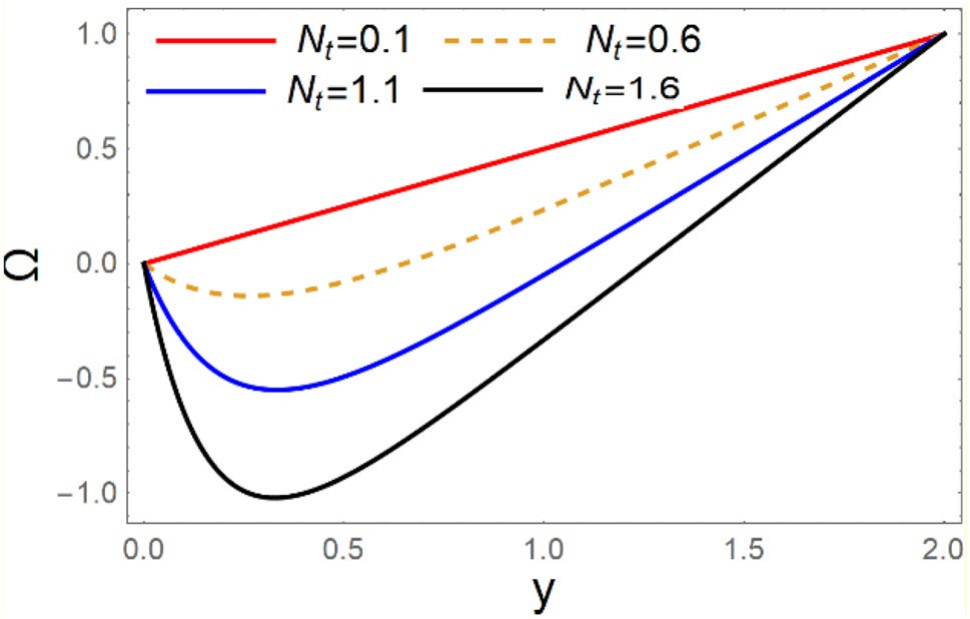
Ω variations for *N*_*t*_.

**Figure 20:**
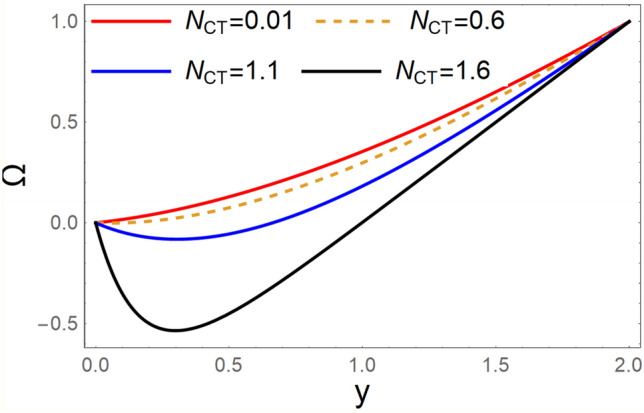
Ω profile for *N*_*CT*_.

**Figure 21:**
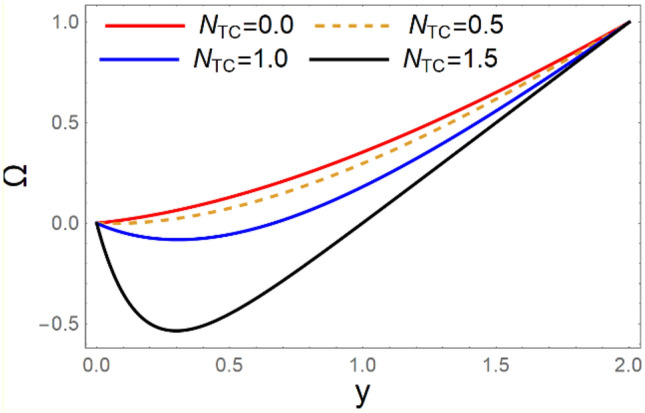
Ω profile for* N*_*TC*_.

**Figure 22:**
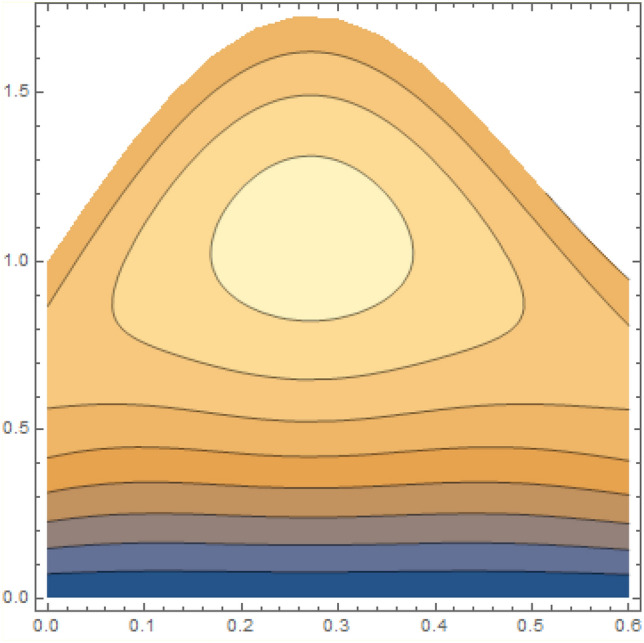
Streamlines of K = 2.5.

**Figure 23:**
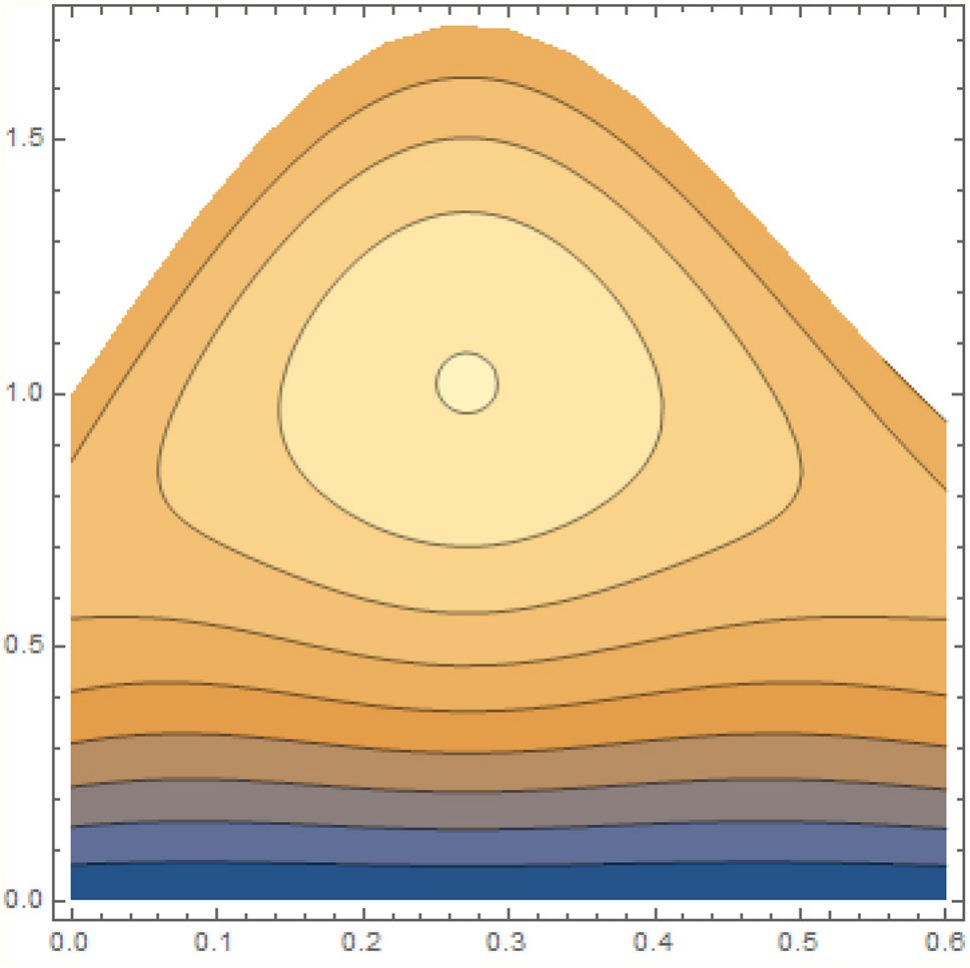
Streamlines of K = 3.5.

**Figure 24:**
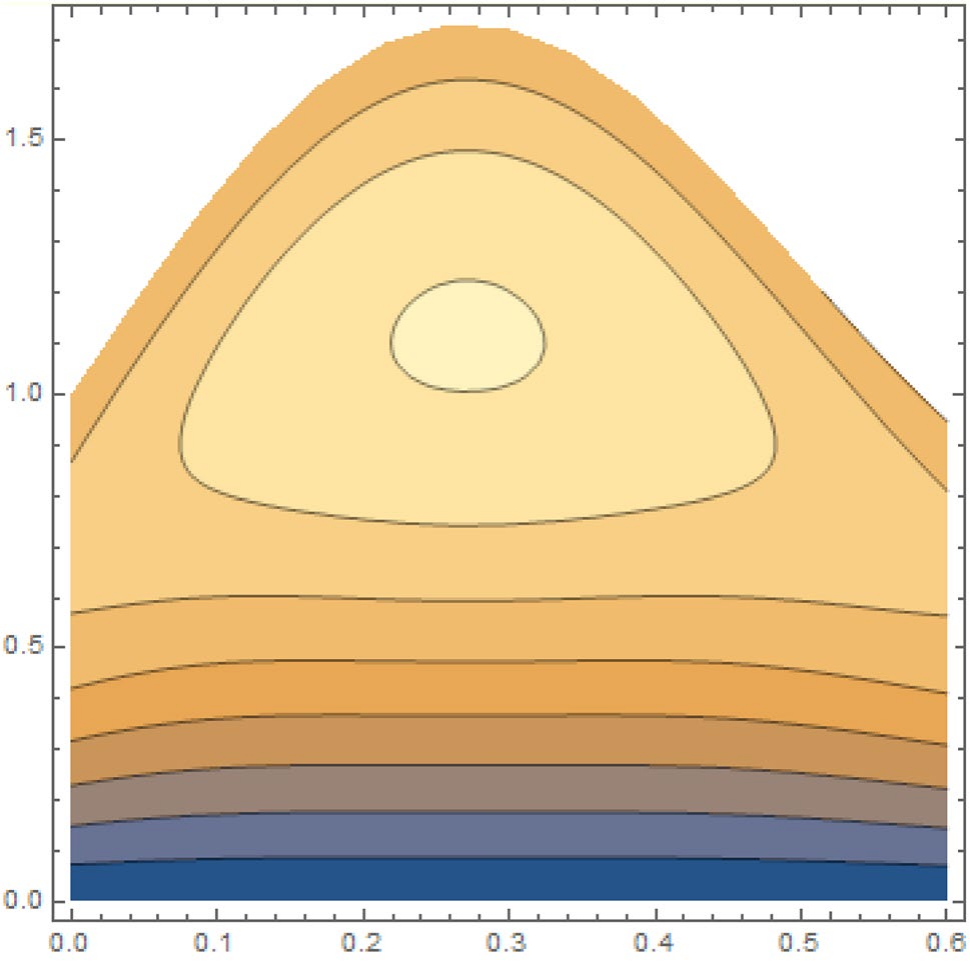
Streamlines of M = 2.0.

**Figure 25:**
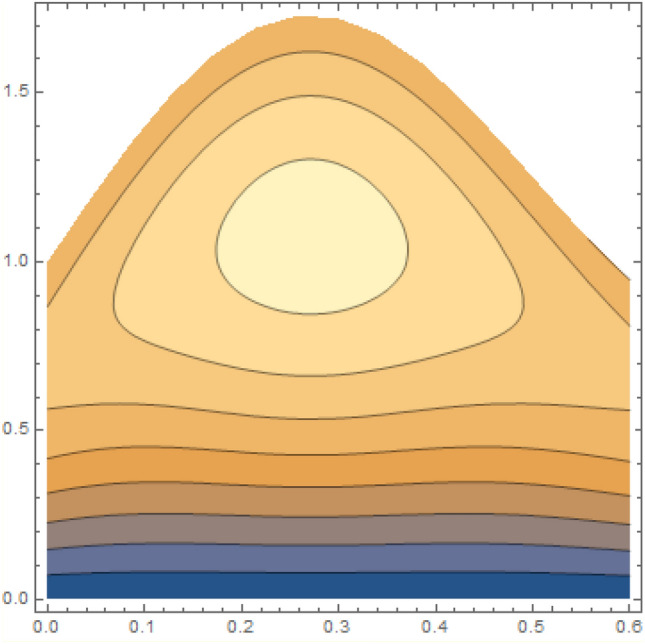
Streamlines of M = 2.2.

**Figure 26:**
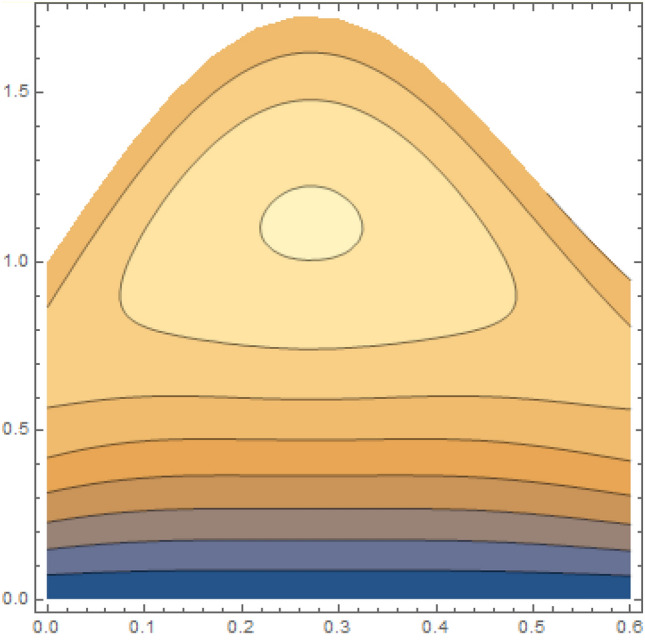
Streamlines of m1 = 0.5.

**Figure 27:**
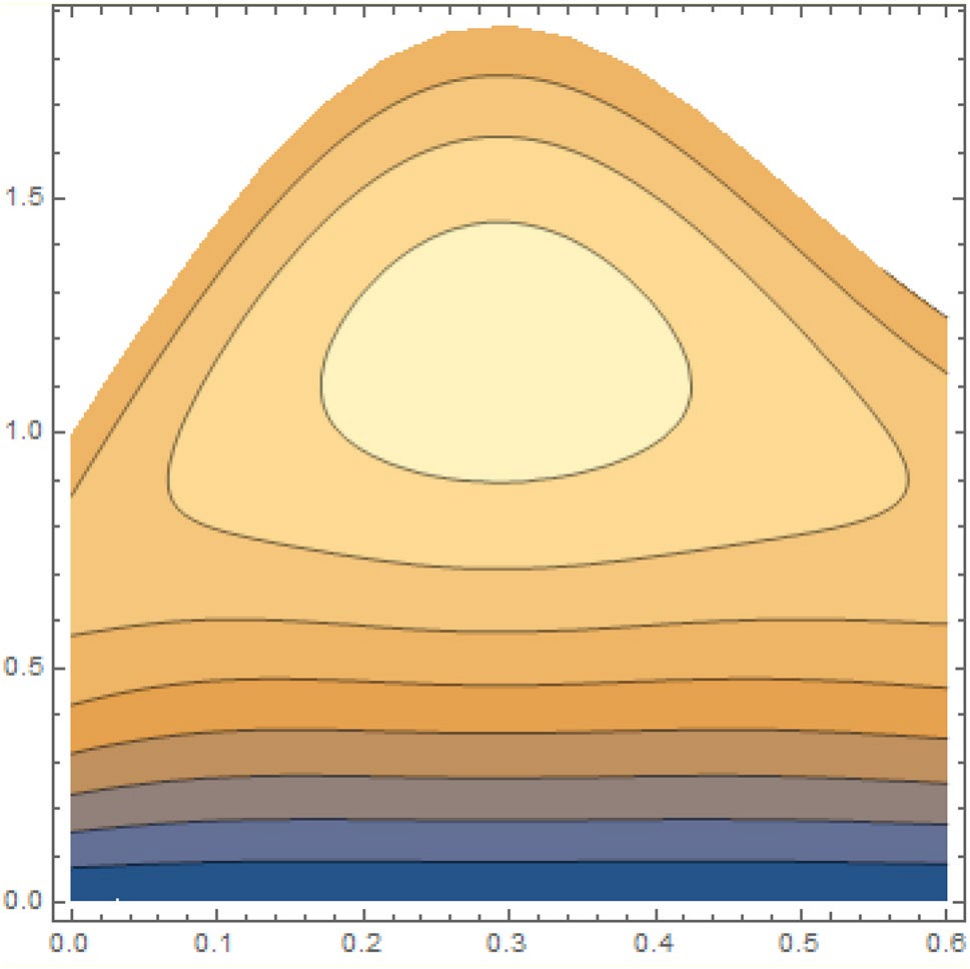
Streamlines of m1 = 1.0.

**Figure 28:**
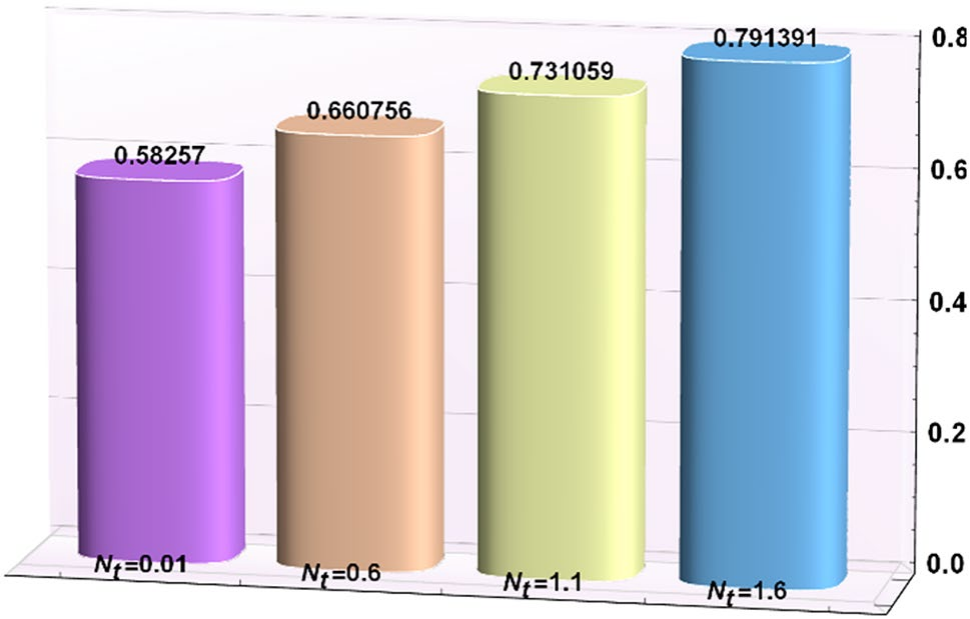
Temperature as function of $N_{t}$.

**Figure 29:**
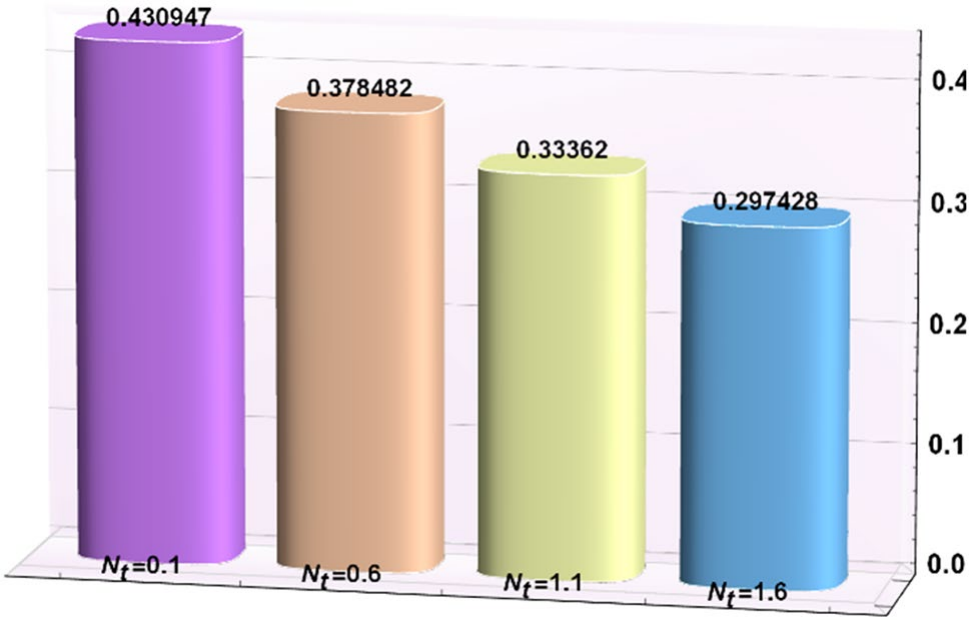
Concentration as function of $N_{t}$.

A magneto couple stress nanofluid flow involving double diffusive convective process has been investigated for peristaltic induced biological problem through non uniform channel. A comprehensive mathematical model is examined for couple stress nanofluid magneto nanofluids. A comparative analysis of current investigation with Afzal et al.^33^ are made in Tables 1, 2 and 3. It is evident from Tables 1, 2 and 3 very small absolute error for temperature, concentration and nanoparticle fraction which validate current study. Computations explored with the help of graphical illustration for several biophysical quantities are portrayed in Figs. 2, 3, 4, 5, 6, 7, 8, 9, 10, 11, 12, 13, 14, 15, 16, 17, 18, 19, 20, 21, 22, 23, 24, 25, 26, 27, 28 and 29 for $$G_{rF} = 1.5,\;G_{rc} = 1.5,\;G_{rt} = 1.5,\;\alpha = 0.3,\;k = 1.4.$$ The impacts of several important variables on the nanoparticle fractions, pressure gradient, pressure rise per wavelength, current density distribution, temperature profiles, solutal concentration, axial induced magnetic field and stream function profiles are displayed in graphical and tabular form. Figures 2 and 3 elucidate the influence of non-uniformity parameter *m1* and thermophoresis parameter *N*_*t*_ on pressure gradient d*p/*d*x*. It is revealed from Fig. 2 that an incremental increase in non-uniformity parameter *m1*, the pressure gradient diminishes asymptotically. From physical point of view, it is natural phenomenon since the channel width is enhanced naturally pressure profile will decrease. From Fig. 3, we observed that as the value of thermophoresis parameter *N*_*t*_ enhances, reduction in the pressure gradient is observed. Physically thermophoresis phenomenon results into higher molecular movement which results into a decay in pressure gradient. Figures 4 and 5 exhibit the impacts of non-uniformity parameter *m1* and thermophoresis parameter *N*_*t*_ on the pumping mechanism. It is observed that pumping phenomenon is greatly influenced by these parameters, pressurize profile is depreciated in region $$y \in [ - 2,1]$$ and is surges in $$y \in [ - 1,4]$$ as non-uniformity parameter *m1* is enhanced. Further, it is reported that pressure rise is compactly surges as thermophoresis parameter *N*_*t*_ is strengthened. Figures 6 and 7 show the effects of magnetic Reynolds number *R*_*m*_ and Hartmann number *M* on the axial induced magnetic field *h*_*x*_. It is quite evident from Fig. 6 that axial induced magnetic field rises in one region $$y \in [ - 1,0]$$ and is repressed in other region $$y \in [0,1].[0,1]$$$$.$$ It is due to the fact that magnetic Reynolds number is the ratio of induction and diffusion, it provides a guess of the relative impacts of induction due to magnetic field due to the dynamics of a conducting medium. Figure 7 shows opposite trends for positive values of magnetic field *M*. Since Hartmann number provides an estimate of the relative significance of drag forces which are generated from magnetic induction and viscous forces during the flow. It may be deduced that axial induced magnetic field distribution is declined initially and then turn around is seen. In order to observe the influences of magnetic Reynolds number *R*_*m*_ and Hartmann number *M* on the current density *J*_*Z*_, we have prepared Figs. 8 and 9. Current density is referred as charge per unit time that flows within some specified region. It is quite evident from the Fig. 8 that the magnetic Reynolds number reinforce current density distribution. Further, as narrated above the magnetic number retard the flow, in the similar manner a declined in the current density distribution is observed as magnetic field is become stronger (Fig. 9). Figures 10, 11, 12 and 13 have been prepared in order to investigate the phenomenon of Brownian motion *N*_*b*_, thermophoresis parameter *N*_*t*_, Soret parameter *N*_*CT*_ and Dufour parameter *N*_*TC*_ on the temperature profile $$\theta .$$ It is noticed that temperature profile enhances with an incremental change in Brownian motion *N*_*b*_, thermophoresis parameter *N*_*t*_, Soret parameter *N*_*CT*_ and Dufour parameter *N*_*TC*_. The qualitative behavior of Brownian motion *N*_*b*_, thermophoresis parameter *N*_*t*_, Soret parameter *N*_*CT*_ and Dufour parameter *N*_*TC*_ on the temperature profile $$\theta$$ is similar. The effects of Brownian motion *N*_*b*_, thermophoresis parameter *N*_*t*_, Soret parameter *N*_*CT*_ and Dufour parameter *N*_*TC*_ on the solutal concentration profile $$\lambda$$ are portrayed in Figs. 14, 15, 16 and 17. We observed that the solutal concentration profile increases with an increase in *N*_*b*_, *N*_*t*_, *N*_*CT*_ and *N*_*TC*_. Figures 18, 19, 20 and 21 depict the effect of Brownian motion *N*_*b*_, thermophoresis parameter *N*_*t*_, Soret parameter *N*_*CT*_ and Dufour parameter *N*_*TC*_ on nanoparticle fraction $$\Omega$$. From Fig. 18, it is noted that as the value of *N*_*b*_ increases, the magnitude of nanoparticle fraction $$\Omega$$ increases in magnitude whereas opposite trend is noted for *N*_*t*_, *N*_*CT*_ and *N*_*TC*_. It is also observed maximum variation in nanoparticle fraction $$\Omega$$ is noted near the lower part of the channel. Trapping phenomenon play a predominant role in all physiological and its significance is explored in Figs. 22, 23, 24, 25, 26 and 27. It has been experienced that the shape of the trapped bolus is significantly reduced with rising values of K. Furthermore, it is quite obvious from Figs. 24, 25, 26 and 27 that size of trapped bolus is enhanced by increasing the values of Hartmann number M and strengthening the non-uniformity parameter m1. A statistical analysis of temperature and concentration profile as function of thermophoresis $$N_{t}$$ is presented in Figs. 28 and 29, it is noticed that temperature profile is lifted with $$N_{t}$$ and reverse phenomenon is seen for concentration profile.

**Table 1:** Comparison of temperature profile solution with $x = 1,\;m1 = 0.5,\;\alpha = 0.5,\;N_{TC} = 0.8,\;N_{b} = 0.1,\;N_{t} = 0.8,\;N_{CT} = 0.8$.

y	Afzal et al.^33^	Current investigation	Absolute error
0	0	0	0
0.2	0.308818	0.308818	$1.27191 \times 10^{ - 9}$
0.4	0.530096	0.530096	$2.6099 \times 10^{ - 9}$
0.6	0.688648	0.688648	$4.13253 \times 10^{ - 9}$
0.8	0.802256	0.802256	$4.94762 \times 10^{ - 9}$
1	0.88366	0.88366	$6.35472 \times 10^{ - 9}$
1.2	0.941988	0.941988	$2.20791 \times 10^{ - 9}$
1.4	0.983782	0.983782	$1.74327 \times 10^{ - 9}$
1.6	1.01373	1.01373	$1.18178 \times 10^{ - 9}$
1.8	1.03519	1.03519	$3.02418 \times 10^{ - 10}$
2	1.05056	1.05056	$4.40595 \times 10^{ - 10}$

**Table 2:** Comparison of concentration profile solution with $x = 1,\;m1 = 0.5,\;\alpha = 0.5,\;N_{TC} = 0.8,\;N_{b} = 0.1,\;N_{t} = 0.8,\;N_{CT} = 0.8$.

y	Afzal et al.^33^	Current investigation	Absolut error
0	0	0	0
0.2	− 0.00705441	− 0.00705441	$1.01753\times {10}^{-9}$
0.4	0.0559234	0.0559234	$2.08791\times {10}^{-9}$
0.6	0.169081	0.169081	$3.30601\times {10}^{-9}$
0.8	0.318195	0.318195	$3.95808\times {10}^{-9}$
1	0.493072	0.493072	$5.08375\times {10}^{-9}$
1.2	0.68641	0.68641	$1.7663\times {10}^{-9}$
1.4	0.892975	0.892975	$1.39458\times {10}^{-9}$
1.6	1.10902	1.10902	$9.45387\times {10}^{-10}$
1.8	1.33185	1.33185	$2.4189\times {10}^{-10}$
2	1.55955	1.55955	$3.52426\times {10}^{-10}$

**Table 3:** Comparison of nanoparticle volume fraction profile solution with $x = 1,\;m1 = 0.5,\;\alpha = 0.5,\;N_{TC} = 0.8,\;N_{b} = 0.1,\;N_{t} = 0.8,\;N_{CT} = 0.8$.

y	Afzal et al.^33^	Current investigation	Absolute error
0	0	0	0
0.2	− 1.27054	− 1.27054	$1.01753\times {10}^{-8}$
0.4	− 1.84077	− 1.84077	$2.08792\times {10}^{-8}$
0.6	− 1.90919	− 1.90919	$3.30603\times {10}^{-8}$
0.8	− 1.61805	− 1.61805	$3.9581\times {10}^{-8}$
1	− 1.06928	− 1.06928	$5.08377\times {10}^{-8}$
1.2	− 0.335902	− 0.335902	$1.76632\times {10}^{-8}$
1.4	0.529746	0.529746	$1.39461\times {10}^{-8}$
1.6	1.49017	1.49017	$9.45423\times {10}^{-9}$
1.8	2.51851	2.51851	$2.41931\times {10}^{-9}$
2	3.59551	3.59551	$3.52471\times {10}^{-9}$

## Conclusion

A mathematical model has been presented for couple stress magneto nanofluids and corresponding equations of motions are handled by applying low Reynolds and long wavelength approximation in viewing the scenario of the physical flow. Computational solution has been explored for nanoparticle volume fraction, solutal concentration and temperature profiles in MATHEMTICA software. The crux of the current study may be interpreted as:

- The pressure gradient decreases by enhancing the values of thermophoresis and non-uniformity parameter.
- Pressure rise shows increasing behavior by strengthening the values of thermophoresis and non-uniformity parameter.
- Current density distribution is becoming strong as the magnetic Reynolds number grows and possesses parabolic profile.
- Temperature profiles is lifted with Soret, Brownian motion, thermophoresis diffusion parameter show opposite behavior for concentration profile.
- The Brownian motion parameter shows inverse relation with nanoparticle fraction Ω.
- The trapping bolus is enhanced with strengthening magnetic field and nonuniformity.

## Data Availability

All the data is provided within the manuscript.

## List of symbols

V, U: Respective velocity component in Y and X directions
$$b$$: Wave amplitude
$a$: Axial distance width
$E$: Induced electric field
$\Theta$: Volume fraction of nanoparticle
$D_{B}$: Brownian diffusion coefficient
$D_{s}$: Solutal diffusion parameter
$R_{m}$: Magnetic Reynolds number
G: Gravitational acceleration
$$T$$: Temperature
$$p$$: Pressure
$b_{0}$: Half width at inlet
c: Propagation of velocity
$$C$$: Solutal concentration
$$D_{TC}$$: Dufour diffusively
$$N_{t}$$: Thermophoresis parameter
$$N_{CT}$$: Soret parameter

## Greek symbols

$$\sigma$$: Electrical conductivity
$$\mu_{e}$$: Magnetic permeability
*α*: Occlusion ratio
*β*_*T*_: Volumetric thermal expansion
$\rho_{p}$: Mass density of nanoparticle
$(\rho c)_{f}$: Heat capacitance of fluid
$$\gamma$$: Solutal concentration
$$\theta$$: Dimensionless temperature
$\mu$: Viscosity of fluid
$\Omega$: Nanoparticle volume fraction
$$\lambda$$: Wave length
$\rho_{f0}$: Fluid density at $T_{0}$
$$\beta_{C}$$: Volumetric expansion
$(\rho c)_{p}$: Heat capacity of nanoparticle
$N_{b}$: Brownian motion parameter
$D_{T}$: Thermophoresis diffusion coefficient
$$N_{TC}$$: Dufour parameter
